# Level of biofilm production by *Staphylococcus aureus* isolates is critical for resistance against most but not all antimicrobial drugs

**DOI:** 10.12669/pjms.38.8.6276

**Published:** 2022

**Authors:** Salah-ud-din, Asad Bashir Awan, Muhammad Mohsin Arshad, Abdul Haque

**Affiliations:** 1 Salah-ud-din, BSc. (Hons.), Department of Health Biotechnology, Akhuwat Faisalabad Institute of Research Science and Technology, (FIRST), Faisalabad, Pakistan; 2 Asad Bashir Awan, M.Phil, Department of Health Biotechnology, Akhuwat Faisalabad Institute of Research Science and Technology, (FIRST), Faisalabad, Pakistan; 3 Muhammad Mohsin Arshad, M.Phil, Department of Health Biotechnology, Akhuwat Faisalabad Institute of Research Science and Technology, (FIRST), Faisalabad, Pakistan; 4 Abdul Haque, PhD, Department of Health Biotechnology, Akhuwat Faisalabad Institute of Research Science and Technology, (FIRST), Faisalabad, Pakistan

**Keywords:** *S. aureus*, Antimicrobial drug resistance, Biofilm production

## Abstract

**Background and Objective:**

Staphylococcal biofilms cause a wide range of acute and chronic infections, both in hospital and community settings across the world. This study explores biofilm forming propensity among *Staphylococcus aureus* clinical isolates from Faisalabad, Pakistan and their association with antimicrobial drug resistance.

**Methods:**

The study was conducted during July to December 2020. The biofilm forming ability of *S. aureus* isolates was assessed by crystal violet staining in 96 well plates. Antimicrobial susceptibility was determined by disk diffusion method against ten antimicrobials representing whole spectrum of antimicrobial drugs.

**Results:**

All the isolates (n=22) produced biofilm; 14 (63.6%) were strong, and 8 (36.4%) moderate biofilm producers. Comparative data were obtained for moderate and strong biofilm producers. Increased biofilm production did not affect azithromycin, clindamycin and mupirocin. However, stronger biofilm production significantly increased resistant isolates in case of augmentin (23.2%), cefoxitin (17.9%), levofloxacin (26.8%), tetracycline (23.2%), vancomycin (14.3%) and trimethoprim (21.4%).

**Conclusions:**

Our findings indicate that the ability to produce large amount of biofilm is an important factor, and *S. aureus* isolates with this ability, do not require acquisition of drug resistance genes from other bacteria. Our study also provides a guideline for selection of antimicrobials which are not adversely affected by level of biofilm production by various strains of *S. aureus*.

## INTRODUCTION

*S. aureus* is a major causative agent for a wide variety of nosocomial (hospital acquired) infectious diseases ranging from benign skin infections like boils, styes, impetigo and localized abscesses which are generally caused by planktonic cells to entrenched/chronic infections such as endocarditis, osteomyelitis, necrotizing pneumonia, furunculosis and food poisoning which are associated with biofilm formation.^1^,^2^

*S. aureus* has been prioritised as the global threat in terms of antibiotic resistance by the World Health Organisation (WHO). The formidable ability of *S. aureus* to reconcile to deviating environmental conditions and to promptly become resistant to virtually all antibiotics accord *S. aureus*, the title of Super Bug.^3^

*S. aureus* aggravate the antibiotic resistance and give rise to outrageous mortality and morbidity due to their proclivity to form biofilms in the wounds of the patients and on the medical devices implanted in the body.^4^ Nearly, 80% of the implanted, prosthetic infections in orthopaedics are due to staphylococcal biofilm.^5^ The biofilm assists the pathogen to neutralize the action of antibiotics and to evade the host immune system resulting in persistent infections.^6^

The biofilm-associated infections are intrinsically strenuous to treat due to a decreased metabolic activity of the cells embedded in biofilms and the shielding nature of a surrounding extracellular matrix (ECM) against environmental factors.^6^,^7^

There are reports of varying effect of biofilms on activity of individual drugs. In this comparative study based on level of biofilm production, we included ten drugs representing all major groups to present a cumulative picture. This study was also aimed to evaluate the effect of biofilm production with respect to increase in the resistance against different antimicrobial groups.

## METHODS

### Collection of Specimens:

The *S. aureus* isolates were collected from samples of patients from local hospitals of Faisalabad, Punjab Pakistan. The samples were collected randomly from all ages and genders.

### Ethics and study duration:

The study was approved by the Institutional Research Ethics Committee of Akhuwat-FIRST with reference number “Akt-FIRST/P21/ethics/05”. The study was conducted during July to December 2020.

### Identification of isolates:

Samples were cultivated in nutrient broth (Oxoid, UK) and on nutrient agar (Oxoid, UK) plates. Isolated colonies were observed under microscope by Gram staining and by colonial morphology. After preliminary identification of *S. aureus* isolates, stocks were made using 25% v/v glycerol in TSB (Tryptic Soy Broth, (Oxoid, UK) and stored at -80°C.

The specimens were revived from the glycerol stock by using TSB according to manufacturer’s instructions. Sub culturing was done on nutrient agar plates. Isolated colonies on nutrient agar were sub cultured on mannitol salt agar to assess mannitol fermentation.

### Coagulase and Catalase Test:

One drop of overnight bacterial suspension was placed on each of the two clean microscopic slides. For coagulase test, one drop of plasma citrate was added and the glass slide was slightly swirled for mixing. Appearance of plasma flocculation indicated coagulase positive isolate. For catalase test, a drop of 3% H_2_O_2_ was added. After mixing with a sterile tooth pick, appearance of bubbles indicated a catalase positive isolate.

### PCR based confirmation of S. aureus:

PCR was performed for the confirmation of *S. aureus* isolates by targeting *nuc* gene, that encodes thermo nuclease and is used for specific detection of *Staphylococci*.^8^ The forward and reverse primers were 5`-GCGATTGATGGTGATACGGT-3` and 5`-AGCCAAGCCTTGACGAACTAAAGC-3` respectively with a product size of 280 bp. For each 25 μl reaction mixture, the final concentrations of reactants were as follows: 1×Taq buffer, 1.5 mM of MgCl_2_, 0.2 mM dNTPs mixture, 0.4 μM of each primer, one unit Taq polymerase with PCR water for volume makeup. DNA template was used as 5 μl from 25 ng/μl stock solution. Thermal cycler conditions were: initial denaturation at 95°C for 5 minutes followed by 30 cycles of: denaturing at 94°C for 1 minute, annealing at 50°C for 1 minute and extension at 72°C for 1 minute, and a final extension at 72°C for 5 minutes. The amplicons were observed by using 1.5% agarose gel electrophoresis.

### Antimicrobial susceptibility testing:

Antimicrobial susceptibility testing was performed using disc diffusion method following the guidelines of Clinical & Laboratory Standards Institute (CLSI, 2020) and European Committee on Antimicrobial Susceptibility Testing (EUCAST breakpoints v.12.0, 2022).^9^ The antimicrobials used were Penicillin G (10 IU), Augmentin (30μg), Cefoxitin (30μg), Levofloxacin (5μg), Tetracycline (30μg), Vancomycin (30μg), Trimethoprim (5μg), Azithromycin (10μg), Mupirocin (200μg), and Clindamycin (2μg).

### Biofilm production studies:

*S. aureus* isolates were grown in sterile Trypticase soy broth (TSB). After overnight incubation, 1:100 dilutions were made in fresh TSB supplemented with 0.2% glucose. The diluted cultures were transferred to a sterile round bottom 96 well plate and incubated at 37°C for 18 hours. In 5 wells, only sterile supplemented medium was added as a negative control. After incubation, the medium was dumped out in bleach solution by turning the plate over. Plates were washed gently in clean water and fingers were moved over them to remove any air bubbles followed by removal of water by turning over the plate. This step was repeated twice to ensure that medium and unattached cells were completely removed which otherwise could give false results.^10^,^11^

After washing, 200μl of 0.1% crystal violet solution was added to each well of microtiter plate and left at RT for 10 minutes. The plates were then washed and water was removed by shaking the plates. This step was repeated three times. The plates were bloated against paper towels and left in upside down position for two hours to dry the wells at room temperature. After drying, 125μl of 30% glacial acetic acid was added to each well and the plates were incubated at room temperature for 15 minutes. The contents of plate were transferred to a new sterile flat bottom micro titer plate. The absorbance value was observed at 650 nm using Microplate Photometer (Multiskan FC, Thermo Scientific). For each isolate, the experiment was performed twice in triplicates per plate.^10^

### Interpretation of level of biofilm production:

For interpretation of biofilm results, the “Mean + 3SD” method was used to find not only the absence and presence of biofilms but also for determination of cut-off values (ODs) to predict weak, moderate and strong biofilm producers (Stepanović et al 2000).^10^,^11^

### Statistical Analysis:

Microsoft Excel 2016 was used to record observations and results as well as to obtain percentage calculations for resistance among the tested isolates.

## RESULTS

### Morphological and biochemical tests:

Out of 32 isolates, 22 were confirmed as Gram positive with typical morphology of *Staphylococci*. All the 22 isolates fermented mannitol as indicated by growth on Mannitol Salt Agar, and were coagulase and catalase positive.

### PCR-based confirmation of S. aureus isolates:

**Fig.1:**
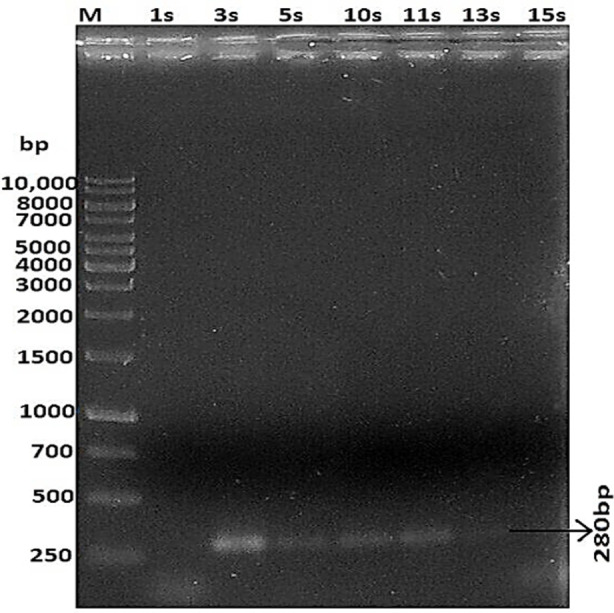
PCR based amplification of nuc gene (280 bp product) M= ladder (GeneRuler 1 kb DNA Ladder, Cat#SM0311), Isolates = 1s, 3s, 5s, 10s, 11s, 13s, 15s

PCR confirmed the presence of thermo nuclease encoding *nuc* gene in 22 *S. aureus* isolates. A single amplification product of 280 bp was obtained in all cases (Fig.1).

### Antimicrobial susceptibility testing:

The resistance showed by *S. aureus* isolates against each antimicrobial is given in Fig.1 while the resistance patterns exhibited by these isolates are given in Table-I. The higher resistance rates were observed against penicillin G (95.5%), augmentin (77.3%) and trimethoprim (63.6%). Clindamycin and Levofloxacin were moderately effective as 54.5% isolates were resistant to both of these drugs. Vancomycin was found the most effective antimicrobial as only 9% isolates were found resistant. Tetracycline and Mupirocin were also found relatively more effective as 27.3% and 31.8% isolates showed resistance against these antimicrobials respectively.

**Table-I:** Antimicrobial resistance against different antimicrobials.

Antimicrobials	Dose per disc	Susceptibility		Resistance	

		Isolates	Percentage	Isolates	Percentage
Augmentin	30 μg	5	22.7%	17	77.3%
Penicliin G	10 IU	1	4.5%	21	95.5%
Cefoxitin	30 μg	14	63.6%	8	36.4%
Levofloxacin	5 μg	10	45.5%	12	54.5%
Tetracyline	30 μg	16	72.7%	6	27.3%
Vancomycin	30 μg	20	90.9%	2	9.1%
Trimthoprim	5 μg	8	36.4%	14	63.6%
Azithromycin	15 μg	11	50.0%	11	50.0%
Mupirocin	200 μg	15	68.2%	7	31.8%
Clindamycin	2 μg	10	45.5%	12	54.5%

μg= micrograms, IU= International units.

### Biofilm Production:

**Fig.2:**
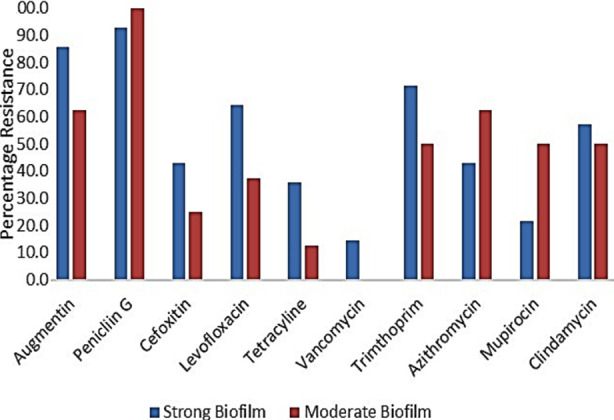
Biofilm formation potential among resistant isolates.

All the 22 *S. aureus* isolates formed biofilms to various degrees; 14 isolates formed strong biofilms and eight produced weak to moderate biofilms. As shown in Table-II, generally, the level of biofilm production is directly related to level of drug resistance in *S. aureus* isolates. *S. aureus* isolates that were resistant to 7 or more drugs (n=5) were all strong producers of biofilm with one exception. Among the *S. aureus* isolates which were resistant to 3-6 drugs, the number of moderate biofilm producers and strong biofilm producers was similar (5 versus 9). Furthermore, it was also observed that the strong biofilm producers exhibited relatively higher resistance against β-lactams, levofloxacin, tetracycline, vancomycin, trimethoprim and clindamycin when compared to moderate biofilm producers (Fig.2). These results strongly suggest a direct role of biofilm production in drug resistance.

**Table-II:** Multidrug resistance related to biofilm production in *S. aureus* isolates.

Group	Resistance against Antimicrobials	Resistance Patterns	Isolates in group	Biofilm
G1	2	P, AZM	2	M
G2	2	LEV, TMP	1	S
G3	3	P, LEV, TMP	1	S
G4	4	AUG, P, TMP, CLI	2	M/S
G5	4	AUG, P, CEF, LEV	3	S
G6	4	P, LEV, TMP, AZM	1	M
G7	5	AUG, P, LEV, TMP, MUP	1	M
G8	5	AUG, P, TET, TMP, CLI	1	S
G9	5	AUG, P, TMP, MUP, CLI	1	M
G10	6	AUG, P, CEF, AZM, MUP, CLI	1	M
G11	6	AUG, P, CEF, LEV, TMP, AZM	1	S
G12	6	AUG, P, TET, V, TMP, CLI	1	S
G13	7	AUG, P, LEV, TMP, AZM, MUP, CLI	1	S
G14	7	AUG, P, CEF, LEV, TMP, AZM, CLI	1	S
G15	7	AUG, P, TET, TMP, AZM, MUP, CLI	1	S
G16	7	AUG, P, TET, V, TMP, AZM, CLI	1	S
G17	8	AUG, P, CEF, LEV, TET, AZM, MUP, CLI	2	M/S

AUG= augmentin 30μg, P= penicillin G 10 IU, CEF= cefoxitin 30μg, LEV= levofloxacin 5μg,

TET= tetracycline 30μg, V= vancomycin 30μg, TMP= trimethoprim 5μg, AZM= azithromycin 15μg,

MUP= mupirocin 200μg, CLI= clindamycin 2μg, M= moderate biofilm, S= strong biofilm

## DISCUSSION

Biofilms have emerged as an important prototype for conceptualizing the relationship between bacteria and their environment. As our knowledge of the biology of biofilms has grown, it has become evident that microorganisms are capable of wholly altering their physiology to cope with the environmental stresses.^12^ As biofilm is one of the major causes of amplifying antibiotic resistance, hence biofilm detection helps in investigating the severity of infection by *S. aureus*.^13^ This study focused on general relationship between biofilm production and multidrug resistance in *S. aureus* isolates, and its effect on recommended antimicrobials.

Penicillin, in general, was ineffective against all isolates. However, in case of augmentin which has clavulanic acid, higher resistance (85.7%) among strong biofilm producers as compared to moderate biofilm producers (62.5%) indicated the involvement of biofilms in reducing the effectiveness. These results are comparable with those reported by Sedlacek and Walker.^14^ They found that 90% or more of the bacteria with biofilms were resistant to augmentin.

In comparison to the resistance (77.3%) against augmentin, the resistance against cefoxitin was significantly lower (36.4%). Same as augmentin, cefoxitin resistance was found higher among strong biofilm producers (43%) as compared to moderate biofilm producers (25%). These results are in agreement with a previous study in which strong biofilm producing *S. aureus* isolates were more resistant to cefoxitin.^15^

Our results show that importance of biofilm production varies according to individual antimicrobials. This aspect has been studied previously on a single or a few antimicrobials. This study provides an overall information about nine antimicrobials representing nine recommended antimicrobial groups for the treatment of *S. aureus* infections.

Levofloxacin is a third generation fluoroquinolone, another front line group of antimicrobials. Our results (Fig.1) indicated that its efficacy was severely affected by increasing amount of biofilm production (37.5% to 64.3%). This observation is supported by Sun and associates who reported that the penetration of ciprofloxacin is significantly reduced through *S. aureus* biofilms.^16^

Tetracycline is a traditional drug which in spite of its age, is still widely prescribed. Our results indicate that increase in biofilm production had significant effect on its efficacy as the difference in resistance level between moderate biofilm producers (12.5%) and strong biofilm producers (35.7%) was as much as 23.2%. Nevertheless, the overall resistance against tetracycline was much lower (27.3%). This observation is in line with that of Stone and associates who concluded that tetracycline has better penetration in biofilms.^17^ However, the effect of stronger biofilm formation in reducing the susceptibility of tetracycline has also been reported by previous studies.^18^

Trimethoprim, a dihydrofolate reductase inhibitor, is the most commonly used antimicrobial often prescribed in combination with a sulfa drug. We found that it was only effective against 36% isolates while the strong biofilm producers exhibited higher resistance (71%) as compared to moderate biofilm producers (50%). This effect of greater biofilm production could be counteracted by using a higher dose (500 μg/ml) only.^19^

Azithromycin and mupirocin were found least affected by increasing amount of biofilm production by *S. aureus* isolates. This is in line with the previous studies where azithromycin and mupirocin have been reported to reduce the biofilm formation potential of *S. aureus* isolates.^20^,^21^

Clindamycin is a derivative of lincomycin, a well-known antibiotic. It was moderately affected by increasing amount of biofilm. Our results have relevance to the findings of Schilcher and coworkers who reported that biofilms produced by some mutants of *S. aureus* are responsive to clindamycin while those of some others are not.^22^

Vancomycin was found to be the most effective antimicrobial as only two isolates showed resistance. Both were strong biofilm producers. It has been previously reported that an increase in the thickness of the cell wall in the biofilms significantly reduces the vancomycin efficacy.^23^ In addition to that, several studies have reported the inhibitory effect of the vancomycin on the biofilm formation when used in synergy with other antimicrobials such as aminoglycosides and rifampin.^24^,^25^

### Limitations of the study:

The molecular mechanisms and variable expression regarding strong biofilm formation were not evaluated in this study.

## CONCLUSIONS

Alarming increase in antimicrobial resistance in bacteria is one of the foremost threats to humanity as acknowledged by WHO. It is a well-known fact that main contributor to this menace is horizontal transfer of drug resistance genes among bacterial populations, but in recent years, biofilm formation has emerged as another potent weapon used by bacteria. In this study we have highlighted the role of biofilm formation in one of the foremost pathogens, *S. aureus*, and shown its direct relationship to resistance towards various antimicrobials. We have also shown that effect of biofilm is not uniform against all antimicrobial drugs and identified the drugs which are least affected, moderately affected and highly affected by the amount of biofilm formed by *S. aureus* isolates.

### Author’s Contribution:

**SA:** Sample collection and Molecular Biology Experiments.

**AB:** Microbiology and Biofilm studies.

**MM:** Sample revival and verification; Data analysis.

**AH:** Concept and overall supervision, he is also accountable for the accuracy of the work.
